# Probiotics *Lactobacillus acidophilus* LA4 and *Lacticaseibacillus paracasei* F5 Alleviate Cognitive Dysfunction in Alzheimer’s Disease Models: A Dual-Screening Study in *Drosophila* and Mice

**DOI:** 10.3390/foods15030429

**Published:** 2026-01-24

**Authors:** Jia Liu, Guoqing Ren, Siyi Niu, Yongshuai Liu, Yuqing Zhao, Zhenou Sun, Qiaomei Zhu, Jixiang Zhang, Yufeng Mao, Zhengqi Liu, Qingbin Guo, Huanhuan Liu

**Affiliations:** 1 State Key Laboratory of Food Nutrition and Safety, College of Food Science and Engineering, Tianjin University of Science and Technology, Tianjin 300457, China; 13954120986@163.com (J.L.); renqing0713@163.com (G.R.); niu_siyi202307@163.com (S.N.); liuyongshuai2026@163.com (Y.L.); zyqrainie@163.com (Y.Z.); zhenousun@tust.edu.cn (Z.S.); qmzhu@tust.edu.cn (Q.Z.); zhangjixiang@tust.edu.cn (J.Z.); 2 Tianjin Institute of Industrial Biotechnology, Chinese Academy of Sciences, 32 West 7th Avenue, Tianjin Airport Economic Area, Tianjin 300308, China; maoyf@tib.cas.cn; 3 Shenzhen Key Laboratory of Food Nutrition and Health, College of Chemistry and Environmental Engineering, Institute for Innovative Development of Food Industry, Shenzhen University, Shenzhen 518060, China; liuzhengqi@szu.edu.cn

**Keywords:** Alzheimer’s disease, probiotics, microbiota-gut–brain axis, neuroinflammation, metabolomics, gut microbiota

## Abstract

Identifying probiotics that modulate the gut–brain axis is vital for non-pharmacological Alzheimer’s disease (AD) therapy. Through a staged screening from transgenic *Drosophila* to a D-galactose/AlCl_3_-induced murine model, *Lactobacillus acidophilus* LA4 and *Lacticaseibacillus paracasei* F5 were prioritized for their ability to improve climbing indices and reduce Aβ deposition and AChE activity. In AD mice, LA4 and F5 significantly ameliorated cognitive deficits and anxiety-like behaviors. Mechanistically, both strains reduced hippocampal Aβ_1–42_ and p-Tau levels, inhibited AChE, suppressed pro-inflammatory cytokines (TNF-α, IL-6, IL-1β), and enhanced antioxidant enzymes (SOD, GSH-Px). 16S rRNA analysis revealed restored Firmicutes/Bacteroidetes ratios and enrichment of SCFA-producers (*Muribaculaceae*, *Dubosiella*). Metabolomics highlighted remodeled purine and arginine pathways, with strain-specific effects on primary bile acid biosynthesis/sphingolipid metabolism (LA4) and butanoate metabolism/nicotinate and nicotinamide metabolism (F5). Consequently, LA4 and F5 alleviate AD pathology by restructuring microbial and metabolic profiles, thereby mitigating neuroinflammation and oxidative stress. These findings confirm the potential of specific probiotics as functional food ingredients for the prevention and adjuvant treatment of neurodegenerative diseases.

## 1. Introduction

Alzheimer’s disease (AD) is a progressive neurodegenerative disorder and the most common form of dementia, affecting over 55 million individuals worldwide [1]. Current therapeutic approaches for AD primarily rely on cholinesterase inhibitors (e.g., donepezil, galantamine) and N-methyl-D-aspartate (NMDA) receptor antagonists (e.g., memantine). Although these agents can improve cognitive dysfunction to a certain extent, they are often associated with severe side effects such as nausea, vomiting, and hypertension [2]. This challenge has necessitated the search for novel intervention strategies that are safe, low in toxicity, and effective.

Neuroscientific research has characterized the core pathologies of AD, which include the deposition of Amyloid-beta (Aβ) protein aggregates forming plaques, the accumulation of hyperphosphorylated tau protein forming neurofibrillary tangles, synaptic dysfunction, and neuronal death [3,4,5]. Notably, the pathological progression of AD is not confined to the central nervous system but is also closely linked to systemic inflammation, metabolic disorders, and gut health [6]. Recent studies have elucidated the intimate connection between the gut microbiota and brain health, termed the “gut–brain axis” [7,8,9,10,11]. The gut microbiota communicates bidirectionally with the brain through various mechanisms, including immune modulation, neuroendocrine signaling, and the vagus nerve. Dysbiosis of the gut microbiota can lead to increased release of inflammatory factors, abnormal neurotransmitter metabolism, and impaired blood–brain barrier integrity, thereby accelerating neuroinflammatory responses and cognitive decline [12,13,14].

Dietary probiotics function as targeted bio-interventions capable of reshaping the gut–brain axis to mitigate neurodegenerative cognitive decline [6,15,16,17,18,19] by strengthening gut barrier function, inhibiting pathogen colonization, modulating immune-inflammatory responses, and enhancing antioxidant capacity. Specific mechanisms include the regulation of neuroinflammation (e.g., inhibiting microglia activation and pro-inflammatory cytokine release), influence on Aβ metabolism (e.g., enhancing Aβ clearance and inhibiting aggregation), mitigation of oxidative stress, protection of synaptic function, and promotion of neurogenesis [20,21]. For instance, *Bifidobacterium breve* A1 has been shown to inhibit hippocampal inflammation and immune response gene expression induced by Aβ [22]. Furthermore, specific *Lactobacilli* strains promote the secretion of extracellular vesicles, which alter the expression of pro- and anti-inflammatory cytokines in the brain and target AMP-activated protein kinase (AMPK) and Protein kinase B (Akt) signaling pathways, ultimately reducing Aβ formation [23].

Both preclinical and clinical studies provide compelling evidence supporting the potential of probiotic interventions in AD. In animal models, oral administration of *Lactobacillus gasseri* MG4247 and *Lacticaseibacillus rhamnosus* MG4644 for three weeks increased the time spent in the target zone during behavioral testing compared to the AD model group, thereby alleviating Aβ-induced cognitive impairment. These probiotics were found to regulate neuroinflammation and neurotoxicity via the Toll-like receptor 4 (TLR4)/Akt pathway and enhance synaptic function by modulating acetylcholine content, acetylcholinesterase (AChE) activity, and the expression of synapse-related proteins [24]. Clinical studies have been equally encouraging; a double-blind controlled trial by Akbari et al. demonstrated that 60 AD patients receiving milk containing multiple probiotic strains for 12 weeks showed a significant improvement in mini-mental state examination scores compared to the control group [25].

As personalized therapy becomes a trend in precision medicine, it is of profound significance to screen for specific effective strains and formulate treatment regimens tailored to host genotypes, baseline gut microecological characteristics, and metabolic phenotypes. Deeply analyzing the specific mechanisms by which novel strains alleviate AD can provide practical microbiome intervention strategies. Consequently, the development of efficient, stable probiotic preparations with clear neuroprotective functions holds significant scientific value and clinical application potential. Based on this premise, the present study focuses on the effects of *Lactobacillus acidophilus*, *Lb. acidophilus*, *Ls. paracasei*, and *Ls. rhamnosus* on Aβ deposition, AChE activity, and cognitive function in AD *Drosophila* and mouse models. A multi-model verification system (comprising both *Drosophila* and mice) was employed to enhance result reliability. Furthermore, multi-omics technologies were integrated to comprehensively evaluate the impact of these probiotics on gut microbiota composition, metabolite profiles, and brain pathological markers. This research aims to provide new strategies for non-pharmacological interventions in AD and to facilitate the development of safe and effective probiotic preparations. These findings possess not only significant clinical translational value but also serve to deepen the scientific understanding of the emerging field of the “gut microbiota–gut–brain axis–neurodegenerative disease” nexus, providing a theoretical basis and experimental evidence for the future prevention and treatment of neurodegenerative disorders.

## 2. Materials and Methods

### 2.1. Materials and Reagents

The probiotic strains *Lb. acidophilus* LA4, *Ls. paracasei* F5, and *Ls. rhamnosus* LR were stored by the Research Center of Food Biotechnology and Future Technology at Tianjin University of Science and Technology. The phylogenetic analysis of probiotics based on 16S rDNA sequences is presented in the Supplementary Materials. Enzyme-linked immunosorbent assay (ELISA) kits (One-step sandwich ELISA) for *Drosophila* Aβ_42_ (Cat. No. TOPEL01908) and AChE (Cat. No. TOPEL01907) were purchased from Beijing Biotopped Biotechnology Co., Ltd. (Beijing, China). ELISA kits (competition method) for mouse Aβ_1–42_ (Cat. No. H296) and AChE (Cat. No. H529-1-1) were obtained from Nanjing Jiancheng Bioengineering Institute (Nanjing, China). Kits (Sandwich ELISA) for mouse tumor necrosis factor-alpha (TNF-α, Cat. No. SEKM-0034), interleukin-6 (IL-6, Cat. No. SEKM-0007), and interleukin-1β (IL-1β, Cat. No. SEKM-0002), as well as biochemical assay kits for glutathione peroxidase (GSH-Px, Cat. No. BC1195), superoxide dismutase (SOD, Cat. No. BC5165), and malondialdehyde (MDA, Cat. No. BC0025), were purchased from Beijing Solarbio Science & Technology Co., Ltd. (Beijing, China). MRS broth was acquired from Qingdao Hope Bio-Technology Co., Ltd. (Qingdao, China). Skimmed milk powder was acquired from Inner Mongolia Yili Industrial Group Co., Ltd. (Inner Mongolia, China). Donepezil Hydrochloride (purity ≥ 98%, *w*/*w*), aluminum chloride (AlCl_3_, purity ≥ 99%, *w*/*w*), and D-galactose (purity ≥ 99%, *w*/*w*) were acquired from Shanghai Macklin Biochemical Co., Ltd. (Shanghai, China).

### 2.2. Probiotic Culture and Lyophilized Powder Preparation

Strains were cultured in MRS broth at 37 °C for 48 h with two consecutive subcultures. The biomass was harvested by centrifugation at 4000× *g* for 10 min at 4 °C and washed twice with sterile physiological saline (9 g/L). Vortex the pellet for 3 s to resuspend the probiotic cells in 2 mL of sterile water. Then, combine the suspension with 4 mL of skimmed milk (prepared by dissolving skim milk powder in sterile water to a final concentration of 0.14 g/mL) and an additional 4 mL of sterile water, and mix thoroughly. Finally, aliquot the mixture into sterile Petri dishes (90 mm × 90 mm). Samples were pre-frozen at −80 °C for 4 h and subsequently lyophilized at −60 °C for 24 h using a freeze-dryer (Christ Alpha 1–4 LD plus, Martin Christ Gefriertrocknungsanlagen GmbH, Osterode, Germany).

### 2.3. Evaluation of Probiotic Efficacy in Drosophila AD Models

Wild-type W^1118^ *Drosophila* and human APP transgenic Aβ_42_ *Drosophila* (Genotype: elav-GAL4; uas-abeta42/CyO) were purchased from Qidong Fangjing Biotechnology Co., Ltd. (Nantong, China). The Aβ_42_ transgenic *Drosophila* line carries a 1.9 kb *KpnI-XhoI* restriction fragment encompassing the full-length open reading frame of human BACE (β-site APP-cleaving enzyme) cDNA, which has been cloned into the *pUAST* plasmid. Flies were maintained at 25 °C with 60–70% relative humidity under a 12 h light/12 h dark cycle. The experimental groups included: Blank Control (BC, W^1118^ females), Model group (AD, Aβ_42_ transgenic females), and intervention groups (LA4, F5, and LR), with 100 flies per group.

The basal medium (800 mL) consisted of 25.29 g sucrose, 50.59 g glucose, 0.58 g anhydrous calcium chloride, 8.48 g agar, 25.75 g yeast powder, 62.16 g cornmeal, 1.60 g potassium sorbate (dissolved in water), 12.0 mL of methylparaben (Dissolve 10 g of methyl paraben in 75 mL of anhydrous ethanol, and then adjust the volume to 100 mL with distilled water.), and distilled water. For the intervention groups, probiotics dissolved in 50 μL of physiological saline were added to the surface of the medium to achieve a viable count of 1 × 10^8^ CFU/mL [26,27].

After 14 days of feeding, the climbing ability was assessed. For each test, 20 flies were placed in an empty vial and allowed to acclimatize for 5 min. During the test, the vial containing fruit flies was positioned vertically with respect to the laboratory bench surface. The vial was tapped gently to knock the flies to the bottom, and the percentage of flies climbing above 10 cm within 10 s was recorded. This procedure was repeated 8 times with 60 s intervals. The Climbing Index (CI) was calculated as the ratio of flies reaching the target height to the total number of flies [28,29].

After 14 days, the *Drosophilas* were starved for 2 h and then anesthetized with nitrogen gas. Rapidly dissect the head on ice using fine forceps, immediately rinse away impurities with pre-cooled physiological saline, and then transfer the sample to a 1.5 mL microcentrifuge tube. Add 2 μL physiological saline per *Drosophil* head and homogenize the sample using a handheld homogenizer on ice [30,31,32,33]. The homogenate was centrifuged at 12,000× *g* for 15 min at 4 °C. The supernatant was collected for the quantification of Aβ_42_ and AChE levels according to the manufacturer’s instructions.

### 2.4. Probiotic Intervention in AD Mouse Model

Specific-pathogen-free (SPF) male C57BL/6J mice (8 weeks old, 20 ± 2 g, *n* = 40) were purchased from Beijing Speifu Laboratory Animal Technology Co., Ltd. (Beijing, China; License No. SYXK (Jinbin) 2023-0007). All animal procedures were approved by the Animal Welfare Ethics Committee of Tianjin University of Science and Technology (Approval No. TUST-2024044) and conducted in strict accordance with ethical guidelines. These mice were raised fed with an ad libitum diet and water in an environment with a temperature of 25 ± 1 °C, in humidity of 50–60%, and in a 12 h light/dark.

Following a one-week acclimatization period, the mice were randomly assigned to five experimental groups (*n* = 8 per group): Blank Control (BC), Model (AD), LA4 intervention (LA4), F5 intervention (F5), and Positive Control (PC). In the first week (following a one-week acclimatization period), AD was induced in all groups except BC via subcutaneous injection of D-galactose (90 mg/kg) into the nuchal region, combined with intragastric administration of AlCl_3_ (20 mg/kg) [34,35,36]. The BC group received physiological saline. From weeks 2 to 10, treatments were administered 1 h after the daily modeling induction. The daily volume for drug administration via oral gavage was 0.1 mL per 20 g of body weight. The LA4 and F5 groups received 1.3 × 10^10^ CFU/kg of the respective probiotic [8,18]. The probiotic, supplied as a lyophilized powder, was reconstituted in sterile physiological saline and adjusted to the desired concentration immediately prior to administration. The PC group received donepezil hydrochloride (5 mg/kg), and the BC and AD groups received physiological saline daily via oral gavage.

#### 2.4.1. Behavioral Testing

After 9 weeks of intervention, the Open Field Test (OFT) and Morris Water Maze (MWM) were conducted to evaluate spatial exploration and learning memory.

MWM apparatus consisted of a circular pool (100 cm diameter) with a submerged platform (10 × 10 cm) and water temperature maintained at 22 ± 1 °C. The protocol included a 6-day acquisition phase (positioning navigation training) followed by a probe trial on day 7. During the acquisition phase, mice were guided to locate the platform to form spatial memory. On day 7, the platform was removed for the spatial exploration test to assess memory retention [11,34,37]. Swimming paths were recorded and analyzed using the DB188 Animal Behavior Analysis System (Beijing Zhishu Duobao Biotechnology Co., Ltd., Beijing, China).

OFT: Mice were acclimatized to the testing room for 30 min. Each mouse was placed in the center of a clean open field apparatus (50 cm × 50 cm × 40 cm) and allowed to explore freely for 3 min [34]. The total distance traveled was tracked by an overhead camera and analyzed using the DB188 system. The apparatus was cleaned with 75% ethanol between tests.

Following behavioral tests, mice were fasted for 12 h and sacrificed. Serum, intestinal contents, and brain tissues were collected.

#### 2.4.2. Biochemical and Histological Testing

After isolating the cerebral cortex and hippocampus from mice, each tissue was homogenized on ice with pre-chilled phosphate-buffered saline (PBS, pH 7.2–7.4) at a weight-to-volume ratio of 1 mg to 9 μL. The homogenates were centrifuged at 3000× *g* for 20 min at 4 °C [38], and the resulting supernatant was collected for subsequent analysis. The supernatant from hippocampal tissue was used to quantify Aβ_1–42_ and AChE levels, while that from the cerebral cortex was assayed for the inflammatory cytokines TNF-α, IL-6, and IL-1β. Oxidative stress markers (GSH-Px, SOD, and MDA) in the cortical tissue were measured following treatment with the corresponding kit-specific extraction buffers. All procedures were performed in strict accordance with the manufacturers’ instructions.

For histological analysis, brain tissues were fixed in 4% (*w*/*v*) paraformaldehyde (24 h, 4 °C). Fixed brain tissue was removed, dehydrated, and embedded in paraffin. The paraffin block was trimmed to an appropriate size, and serial sections (4 μm thick) were cut and dried in a 60 °C oven for further processing.

Hematoxylin and Eosin (H&E) staining: Deparaffinization was performed by two consecutive xylene treatments. Sections were then rehydrated through a descending ethanol series (100%, 95%, 80%, 75%) and stained with hematoxylin for 5 min. After rinsing with distilled water, sections were differentiated in 1% hydrochloric acid–ethanol for 10 s, followed by a 5 min water rinse. Subsequently, sections were counterstained with 0.5% eosin for 3 min. Excess dye was removed, and sections were dehydrated through an as-cending ethanol series (75%, 80%, 95%, 100%), 1 min per step, followed by two 5 min xylene clearing steps. After mounting, the sections were examined under a light microscope (Nikon Eclipse CI, Tokyo, Japan) for image acquisition and quantitative analysis [39,40].

Immunofluorescence staining for phosphorylated Tau (p-Tau): Following antigen retrieval and serum blocking, the primary antibody (p-Tau antibody, 1:200 dilution; Catalog No. BS-3446R, Beijing Biosynthesis Biotechnology Co., Ltd., Beijing, China), was applied and incubated overnight at 4 °C. After three washes with PBS, a fluorescent secondary antibody (goat anti-rabbit IgG conjugated to FITC, 1:200 dilution; Cat. No. A0562, Shanghai Biyun Tian Biotechnology Co., Ltd., Shanghai, China) was added to fully cover the tissue and incubated for 1 h at room temperature in the dark. Nuclei were counterstained with 4′,6-Diamidino-2-phenylindole (DAPI) for 5 min [41,42]. Fluorescence images were acquired using a panoramic digital slide scanner (3DHISTECH Kft., Budapest, Hungary) and quantitative analysis was performed using ImageJ 1.53 software [43].

#### 2.4.3. Gut Microbiota Testing

Total genomic DNA was extracted from colonic contents using the YH-feces FastPure Stool DNA Isolation Kit (T10-100 MJYH, Shanghai Majorbio Bio-pharm Technology Co., Ltd., Shanghai, China). DNA concentration was quantified using a NanoDrop 2000 spectrophotometer (Thermo Fisher Scientific Inc., Waltham, MA, USA). The hypervariable region V3–V4 of the bacterial 16S rRNA gene were amplified with primer pairs 338F (5′-ACTCCTACGGGAGGCAGCAG-3′) and 806R (5′-GGACTACHVGGGTWTCTAAT-3′) byT100 Thermal Cycler PCR thermocycler (BIO-RAD, Hercules, CA, USA). The PCR reaction mixture including 4 μL 5× Fast Pfu buffer, 2 μL 2.5 mM dNTPs, 0.8 μL each primer (5 μM), 0.4 μL Fast Pfu polymerase, 10 ng of template DNA, and ddH2O to a final volume of 20 µL. The PCR product was extracted from 2% agarose gel and purified using the PCR Clean-Up Kit (YuHua, Shanghai, China) according to manufacturer’s instructions and quantified using Qubit 4.0 (Thermo Fisher Scientific, USA). PCR products were purified, then libraries were constructed using the NEXTFLEX Rapid DNA-Seq Kit (Bioo Scientific, Cat. No. NOVA-5144, Austin, TX, USA), and sequenced on an Illumina Nextseq2000 platform (Illumina, San Diego, CA, USA). Raw FASTQ files were de-multiplexed using an in-house perl script, and then quality-filtered by fastp version 0.19.6 [44] and merged by FLASH version 1.2.7 [45]. The optimized sequences were clustered into operational taxonomic units (OTUs) using UPARSE 11.0.667 [46] at a 97% sequence similarity threshold, and the most abundant sequence within each OTU was designated as its representative sequence.

#### 2.4.4. LC-MS Untargeted Metabolomics

Approximately 50 ± 5 mg of colonic content was accurately weighed into a 2 mL microcentrifuge tube. Subsequently, 400 µL of extraction buffer-comprising four internal standards (palmitoyl-L-carnitine-(N-methyl-d_3_), cholic acid-2,2,4,4-d_4_, L-phenylalanine-d_5_, and 2-chloro-L-phenylalanine)-in extraction solvent (methanol: water = 4:1, *v*/*v*) was added. The mixture was homogenized using a cryogenic tissue grinder for 6 min at −10 °C and 50 Hz, followed by low-temperature ultrasonication for 30 min at 5 °C and 40 kHz. Samples were then incubated at −20 °C for 30 min to allow precipitation, and centrifuged at 13,000× *g* for 15 min at 4 °C. The resulting supernatant was transferred to an HPLC vial containing a glass insert for analysis. Additionally, 20 µL of supernatant from each sample was pooled to generate a quality control (QC) sample for batch monitoring. Metabolomic analysis was performed using a UHPLC-Triple TOF 6600 system (AB Sciex LLC, Redwood City, CA, USA) equipped with an ACQUITY UPLC HSS T3 column (100 mm × 2.1 mm i.d., 1.8 μm; Waters, Milford, MA, USA). The mobile phases consisted of (A) water and acetonitrile (95: 5, *v*/*v*) with 0.1% (*v*/*v*) formic acid and (B) acetonitrile, isopropanol, and water (47.5: 47.5: 5, *v*/*v*/*v*), with 0.1% (*v*/*v*) formic acid. The gradient elution program is as follows: The initial mobile phase B is set at 0% (expressed as the volume percentage of mobile phase B in the total mobile phase) and maintained for 0.2 min. It is then linearly increased to 25% over the interval from 0.2 to 3.0 min, followed by a further linear increase to 100% between 3.0 and 9.0 min. The composition is held at 100% from 9.0 to 10.0 min to ensure complete elution of strongly retained analytes. At 10.1 min, the proportion of mobile phase B is rapidly reduced to 0% and maintained at this level until 12.0 min to allow full re-equilibration of the chromatographic system prior to the next injection. The flow rate was 0.40 mL/min, temperature 45 °C, and injection volume 10 μL. Mass spectrometry was performed in positive and negative electrospray ionization (ESI) modes (*m*/*z* 50–1200). Source parameters were: ion source gas1 at 50 psi, ion source gas2 at 50 psi, curtain gas at 35 psi, spray voltage +5500/−4500 V, and source temperature 500 °C. Data were processed using Progenesis QI v3.0 (Waters Corporation), and metabolites were putatively annotated against HMDB and METLIN databases (mass error < 10 ppm) [47,48,49].

#### 2.4.5. Statistical Analysis

Statistical analyses were performed using GraphPad Prism 10.0.0 (GraphPad Software, Inc., San Diego, CA, USA). Data are expressed as mean ± standard deviation (SD). Differences between groups were analyzed using one-way analysis of variance (ANOVA) followed by Tukey’s post hoc test for multiple comparisons.

## 3. Results

### 3.1. Effects of Probiotics on Motor Function and Biochemical Indices in the AD Drosophila Model

**Figure 1:**
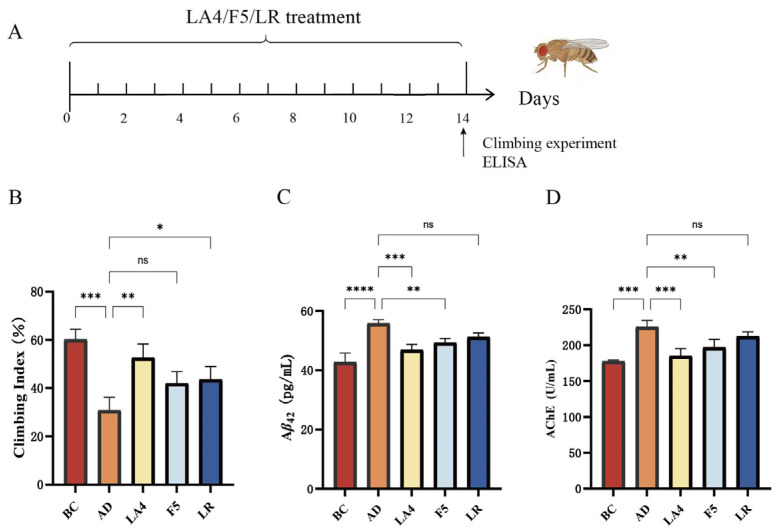
Screening of probiotic strains for alleviating AD symptoms in a *Drosophila* model. (**A**) Experimental timeline for *Drosophila* culture and probiotic treatment. (**B**) Climbing index representing motor coordination (*n* = 100). (**C**) Aβ_42_ levels in *Drosophila* heads. (**D**) AChE activity. BC: Blank Control; AD: Model group; LA4: *Lb. acidophilus* LA4; F5: *Ls. paracasei* F5; LR: *Ls. rhamnosus* LR. Data are presented as mean ± SD. ^ns^ *p* > 0.05, * *p* < 0.05, ** *p* < 0.01, *** *p* < 0.001, and **** *p* < 0.0001.

This study initially utilized a transgenic Aβ_42_ *Drosophila* model to evaluate the intervention effects of *Lb. acidophilus* LA4, *Ls. paracasei* F5, and *Ls. rhamnosus* LR on AD (Figure 1A). The results of the climbing assay (Figure 1B) indicated that the climbing index of the AD model group was significantly reduced compared to the BC group (31 ± 5.4% vs. 60 ± 4.2%, *p* < 0.001, *n* = 100), signifying impaired motor coordination. Intervention with LA4 significantly ameliorated this phenotype (53 ± 5.7%, *p* < 0.01 vs. AD). While F5 (42 ± 4.8%) and LR (44 ± 5.3%) exhibited trends of improvement, statistical significance was achieved only in the LR group (*p* < 0.05 vs. AD).

The levels of Aβ_42_ and AChE activity in *Drosophila* brain tissues are presented in Figure 1C,D. In the AD model group, cerebral Aβ_42_ content was significantly elevated compared to the BC group (28 ± 3.4 ng/mg vs. 15 ± 2.5 ng/mg, *p* < 0.0001), confirming the successful establishment of the AD pathological model. Treatment with LA4 and F5 significantly reduced Aβ_42_ deposition (19 ± 3.3 ng/mg, *p* < 0.001; and 22 ± 3.4 ng/mg, *p* < 0.01 vs. AD, respectively). Although the Aβ_42_ level in the LR group (24 ± 3.2 ng/mg) was lower than that in the AD group, the difference was not statistically significant (*p* > 0.05). Regarding cholinergic system function, AChE activity was significantly upregulated in the AD group (100 ± 13 U/mg vs. 55 ± 11 U/mg in BC, *p* < 0.001). Conversely, interventions with LA4 (63 ± 12 U/mg, *p* < 0.001 vs. AD) and F5 (75 ± 13 U/mg, *p* < 0.01 vs. AD) effectively reversed this abnormality.

Integrated analysis of behavioral and biochemical indices demonstrated that LA4 exhibited the most pronounced effects in improving motor function, reducing Aβ_42_ deposition, and modulating AChE activity. Notably, the efficacy of LA4 in Aβ_42_ clearance (31.5% reduction) was superior to that of F5 (22.9%) and LR (15.9%), suggesting the existence of strain-specific neuroprotective mechanisms.

### 3.2. Mouse Behavioral Analysis

**Figure 2:**
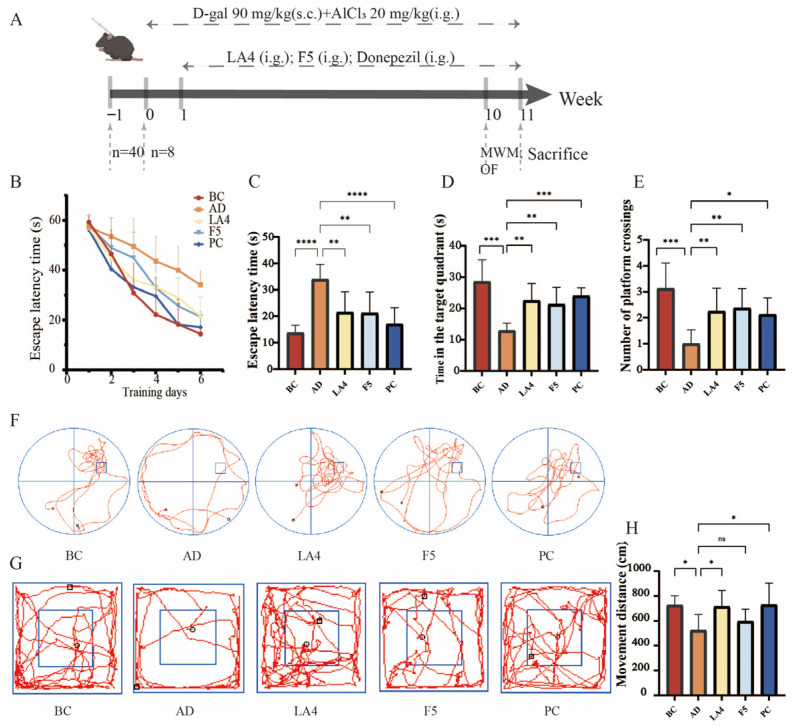
Evaluation of the neuroprotective capabilities of probiotics in D-galactose/AlCl_3_ induced AD mice. (**A**) Schematic overview of the experimental design, including AD induction and probiotic administration timeline. Mice received equal doses of the drug via either subcutaneous injection (s.c.) or intragastric gavage (i.g.). (**B**) Escape latency during the 6-day acquisition phase of the MWM. (**C**) Escape latency on day 6 of the MWM. (**D**) Time spent in the target quadrant during the probe trial. (**E**) Number of platform crossings during the probe trial. (**F**) Representative swimming trajectories in the MWM. (**G**) Representative movement tracks in the OFT experiment. (**H**) Total movement distance in the OFT. Data are presented as mean ± SD (*n* = 8). ^ns^ *p* > 0.05,* *p* < 0.05, ** *p* < 0.01, *** *p* < 0.001, and **** *p* < 0.0001.

Based on the preliminary screening results in the *Drosophila* model, the neuroprotective efficacy of the selected strains, *Lb. acidophilus* LA4 and *Ls. paracasei* F5, was further systematically evaluated in an AD mouse model. The cognitive improvement effects of these probiotics were quantified using the MWM and OFT, focusing on the restoration of spatial learning and memory and the alleviation of anxiety-like behaviors (Figure 2A).

As shown in Figure 2B, during the acquisition phase (positioning navigation training) of the MWM, the learning curves exhibited distinct inter-group differences. As training progressed, mice in the BC group demonstrated normal learning acquisition, characterized by a linear decrease in escape latency. Conversely, the AD group displayed significant spatial learning deficits; starting from day 3 of training, their latency to locate the submerged platform was significantly longer than that of the BC group (*p* < 0.01). However, probiotic intervention effectively mitigated these cognitive deficits. Specifically, compared to the AD group, the positive drug (donepezil hydrochloride) group showed a significant reduction in latency starting from day 2 (*p* < 0.05), while the LA4 group exhibited significant improvement beginning on day 5 (*p* < 0.05). By day 6, the escape latency of the AD group remained elevated (34 ± 5.6 s), significantly higher than that of the BC group (14 ± 2.9 s, *p* < 0.0001). Notably, both the LA4 group (22 ± 7.8 s, *p* < 0.01) and the F5 group (21 ± 7.8 s, *p* < 0.01) showed a marked reduction in latency, with an improvement degree comparable to that of the PC group (17 ± 6.2 s) (Figure 2C). These results indicate that both probiotic strains effectively protected spatial learning acquisition in AD mice.

In the probe trial, where the platform was removed, memory retention capabilities were further assessed. The time spent in the target quadrant (13 ± 2.4 s) and the number of crossings over the original platform location (1.0 ± 0.53 times) in the AD group were significantly lower than those in the BC group (29 ± 6.9 s, *p* < 0.001; and 3.1 ± 0.99 times, *p* < 0.0001, respectively). Furthermore, swimming trajectories revealed random wandering patterns lacking purposefulness (Figure 2F), confirming severe memory retrieval impairments in the model mice. Probiotic intervention significantly reversed this memory damage: the time spent in the target quadrant was extended in the LA4 and F5 groups to 22 ± 5.4 s and 21 ± 5.4 s, respectively (both *p* < 0.01 vs. AD). Similarly, the number of platform crossings was significantly restored (2.3 ± 0.89 times, *p* < 0.05; and 2.4 ± 0.74 times, *p* < 0.01 vs. AD, respectively) (Figure 2D,E). These data suggest that probiotic intervention enhances the consolidation and retrieval of spatial memory in AD mice.

Additionally, the OFT result revealed the potential regulatory effects of probiotics on behavioral and psychological symptoms of dementia. The total movement distance in the AD model group (520 ± 130 cm) was significantly lower than that of the BC group (730 ± 76 cm, *p* < 0.05). Trajectory analysis (Figure 2G) indicated pronounced thigmotaxis in AD mice, with activity confined primarily to the periphery due to anxiety, and minimal exploration of the central area. This suggests that AD pathology induced anxiety-like behaviors or reduced exploratory motivation. Intervention with LA4 significantly restored autonomous activity, increasing the total movement distance to 720 ± 130 cm (*p* < 0.05 vs. AD) and significantly enhancing exploration in the central zone while reducing peripheral stagnation. In contrast, although the F5 group showed an improving trend (600 ± 97 cm), it did not reach statistical significance (Figure 2H).

In summary, both *Lb. acidophilus* LA4 and *Ls. paracasei* F5 significantly ameliorated spatial learning and memory impairments in AD mice. Notably, *Lb. acidophilus* LA4 exhibited superior neuroprotective potential in alleviating AD-associated anxiety-like behaviors and restoring exploratory activity, suggesting that distinct strains may possess specific mechanisms for modulating higher-order neural functions.

### 3.3. Regulatory Effects of Probiotics on Neuropathological Markers, Neuroinflammation, and Oxidative Stress Levels in the Brains of AD Mice

To elucidate the molecular mechanisms underlying the improvement of AD-like behavioral phenotypes by lactic acid bacteria, this study further quantified key neuropathological markers, inflammatory mediators, and oxidative stress indices in mouse brain tissues.

**Figure 3:**
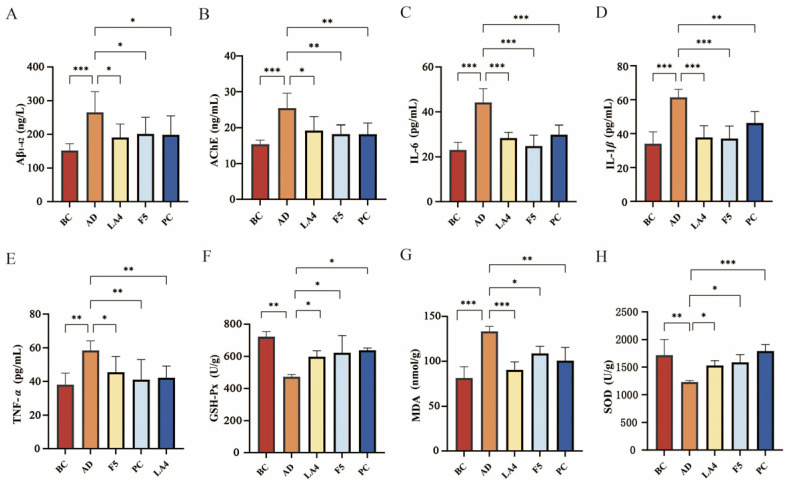
Effects of probiotics on neuropathological markers, neuroinflammation, and oxidative stress in mouse brains. (**A**) Aβ_1–42_ levels in the hippocampus. (**B**) AChE activity. (**C**) IL-6 levels. (**D**) IL-1β levels. (**E**) TNF-*α* levels. (**F**) GSH-Px activity. (**G**) MDA levels. (**H**) SOD activity. Data are presented as mean ± SD. * *p* < 0.05, ** *p* < 0.01, and *** *p* < 0.001.

The detection of Aβ plaques, a specific biomarker for AD, is considered a definitive diagnostic criterion [4]. ELISA results (Figure 3A) indicated that Aβ_1–42_ levels in the hippocampus of the AD mice were abnormally elevated to 270 ± 61 ng/L, significantly higher than those in the BC group (150 ± 21 ng/L, *p* < 0.0001), confirming the successful establishment of the pathological model. Interventions with *Lb. acidophilus* LA4 and *Ls. paracasei* F5 significantly reversed this pathological alteration. The Aβ_1–42_ content was reduced to 190 ± 39 ng/L (*p* < 0.001 vs. AD) and 200 ± 49 ng/L (*p* < 0.01 vs. AD), respectively.

The use of cholinesterase inhibitors in AD treatment is predicated on the critical role of the cholinergic system in cognition [50], and the assessment of AChE activity serves as an indirect measure of cholinergic system impairment. As shown in Figure 3B, AChE activity in the brains of the AD group was significantly upregulated to 25 ± 4.1 ng/mL (*p* < 0.001 vs. BC), suggesting compromised cholinergic transmission. Probiotic intervention effectively inhibited AChE activity, with both the LA4 group (15 ± 1.2 ng/mL, *p* < 0.05 vs. AD) and the F5 group (18 ± 2.5 ng/mL, *p* < 0.05 vs. AD) restoring levels to near-normal values. This indicates that probiotics contribute to maintaining the metabolic homeostasis of neurotransmitters.

Neuroinflammation is a key driver accelerating neurodegeneration in AD. As shown in Figure 3C–E, the levels of pro-inflammatory cytokines IL-6, TNF-*α*, and IL-1β in the cerebral cortex of AD mice were all significantly elevated (all *p* < 0.01 vs. BC), indicating a distinct inflammatory state within the brain tissue. Treatment with both LA4 and F5 significantly reduced the concentrations of these inflammatory factors. For instance, in the LA4 group, IL-6 levels decreased to 28 ± 2.5 pg/mL (*p* < 0.001 vs. AD), TNF-*α* to 42 ± 6.9 pg/mL (*p* < 0.05 vs. AD), and IL-1β to 38 ± 7.1 pg/mL (*p* < 0.001 vs. AD). These findings suggest that probiotics may exert neuroprotective effects by suppressing neuroinflammatory responses.

Mice in the AD group exhibited marked oxidative stress, evidenced by significantly increased MDA levels (130 ± 5.7 nmol/g, *p* < 0.001 vs. BC) and impaired GSH-Px and SOD activities (*p* < 0.01 vs. BC). However, treatment with LA4 and F5 mitigated these pathological changes. Specifically, both probiotics significantly lowered MDA concentrations and replenished antioxidant enzyme defenses (all *p* < 0.05 vs. AD), thereby re-establishing redox homeostasis in the brain (Figure 3F–H).

In summary, *Lb. acidophilus* LA4 and *Ls. paracasei* F5 not only alleviated the cerebral Aβ burden and protected cholinergic function but also exerted synergistic neuroprotective effects via multiple targets by blocking neuroinflammatory cascades and enhancing the endogenous antioxidant defense system.

### 3.4. H&E Staining and Immunofluorescence Analysis

**Figure 4:**
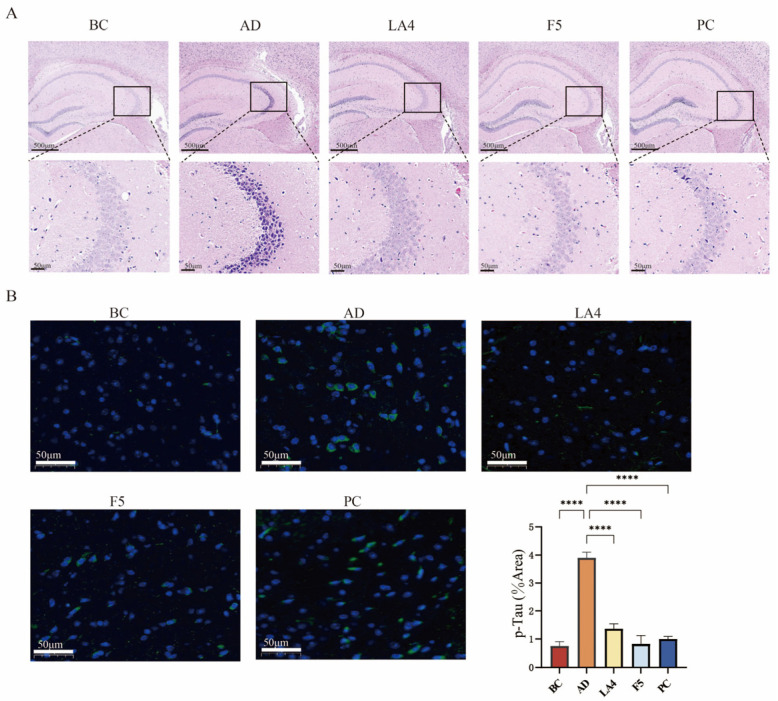
Histopathological evaluation and Tau phosphorylation analysis. (**A**) H&E staining of the hippocampus. The upper row shows the whole hippocampus (4× magnification, scale bar = 200 μm); the lower row shows the CA3 region (20× magnification, scale bar = 50 μm). Boxes indicate the magnified areas. (**B**) Representative immunofluorescence images of p-Tau (green) and DAPI (blue) in the cerebral cortex (20× magnification, scale bar = 50 μm), along with quantitative analysis of the p-Tau positive area percentage. Data are presented as mean ± SD. **** *p* < 0.0001.

The results of H&E staining are presented in Figure 4A. In the BC group, the hippocampal structure appeared normal, characterized by a high density of neurons, an orderly arrangement, and regular somatic morphology. Conversely, the AD model group exhibited severe structural abnormalities in the hippocampus, including a reduced number of neurons, disordered arrangement, and extensive neuronal degeneration. Degenerated neurons displayed shrunken (pyknotic), hyperchromatic cell bodies with irregular shapes. Intervention with *Lb. acidophilus* LA4 resulted in only mild hippocampal abnormalities; the neuronal density was high, with cells arranged in a tight and orderly manner, exhibiting regular morphology, and neuronal degeneration was observed only occasionally. Similarly, the PC group showed mild structural abnormalities, maintaining a high number of neurons with an orderly arrangement, although a small number of degenerated neurons with shrunken, hyperchromatic cell bodies were present.

Hyperphosphorylation of Tau protein disrupts the neuronal axonal transport system, leading to the aggregation of hyperphosphorylated Tau into neurofibrillary tangles (NFTs). The deposition of NFTs is positively correlated with cognitive decline and represents one of the hallmark pathological features of AD and other cognitive disorders [51]. Immunofluorescence staining results demonstrated the distribution of p-Tau in the cerebral cortex, labeled with green fluorescence, while cell nuclei were counterstained with DAPI (blue fluorescence). The BC group exhibited extremely weak, punctate fluorescence. In contrast, the AD group displayed a large number of fluorescent granules aggregated in the perinuclear region of neurons. The groups treated with LA4, F5, and the positive control exhibited fine granular structures, suggesting that these interventions effectively inhibited Tau hyperphosphorylation and reduced its aggregation (Figure 4B).

Quantitative analysis of the fluorescence area revealed that the proportion of p-Tau positive area in the AD group increased to 3.9 ± 0.20%, which was significantly higher than that in the BC group (0.76 ± 0.15%, *p* < 0.0001). Interventions with LA4 (1.4 ± 0.18%) and F5 (0.82 ± 0.31%) significantly suppressed this abnormal accumulation (*p* < 0.0001 vs. AD), demonstrating that LA4 and F5 can mitigate AD progression by inhibiting the hyperphosphorylation of Tau protein.

### 3.5. Gut Microbiota Analysis

**Figure 5:**
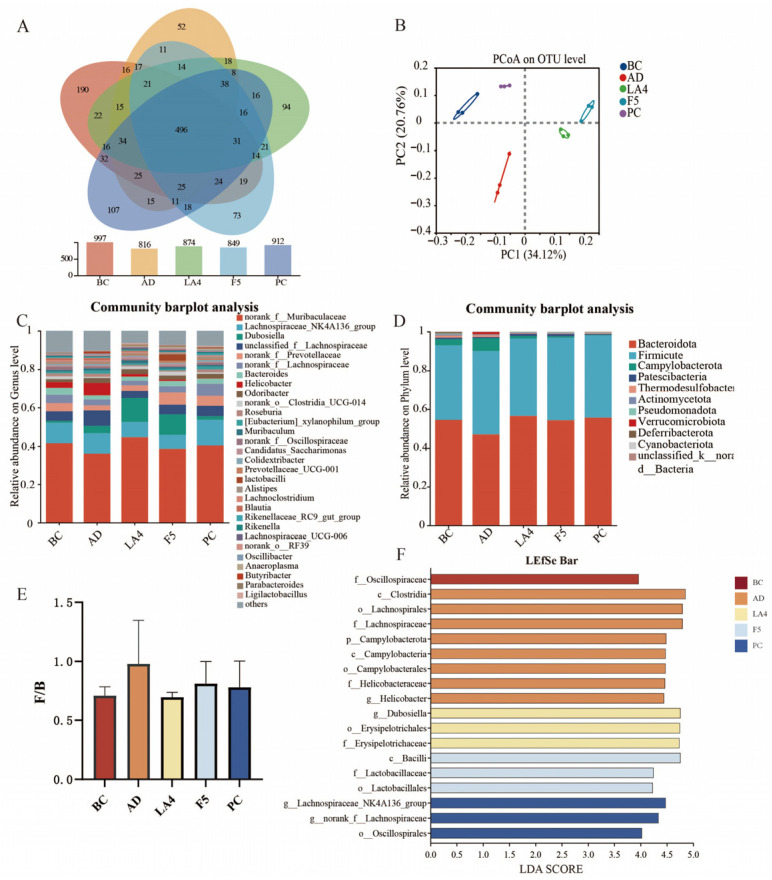
Modulation of gut microbiota composition by probiotics in AD mice. (**A**) Venn diagram showing the number of shared and unique OTUs among groups. (**B**) Principal Coordinate Analysis (PCoA) score plot based on UniFrac distances. (**C**) Relative abundance of gut microbiota at the genus level. (**D**) Relative abundance of gut microbiota at the phylum level. (**E**) The ratio of *Firmicutes* to *Bacteroidetes* (F/B). (**F**) Linear discriminant analysis Effect Size (LEfSe) analysis identifying differentially abundant taxa (LDA score > 3.8) across groups.

As a critical mediator of the gut–brain axis, the gut microbiota and its structural alterations serve as a vital entry point for elucidating the mechanisms of probiotic action. To investigate the effects of probiotic intervention on the gut microbiota structure in mice, the composition of the gut microbiome across all groups was compared using sequencing analysis. Principal Coordinate Analysis (PCoA) based on UniFrac distances revealed distinct clustering among the five experimental groups (BC, AD, LA4, F5, and PC) [52]. The first and second principal coordinates (PCoA1 and PCoA2) accounted for 34.12% and 20.76% of the total variance, respectively (Figure 5B), indicating significant shifts in microbial community structure.

Detailed analysis at the genus level (Figure 5C) indicated a significant reduction in the relative abundance of beneficial short-chain fatty acids (SCFAs)-producing genera in the AD model group. Specifically, the abundance of *Muribaculaceae* decreased to 36.1%, and *Dubosiella* declined to 3.8%. This depletion may exacerbate intestinal barrier injury and systemic inflammation. Following probiotic intervention, the abundance of these beneficial genera was significantly restored. In the LA4 group, *Muribaculaceae* and *Dubosiella* levels recovered to 44.5% and 12.6%, respectively; similarly, in the F5 group, their levels reached 38.6% and 10.7%, respectively. These findings suggest the potential of these strains to maintain intestinal homeostasis through immunomodulation. Concurrently, the abundance of *Helicobacter*, a genus associated with pathogenicity (including *H. pylori*), was abnormally elevated to 6.5% in the model group. Both probiotic intervention groups significantly inhibited this increase, reducing levels to 2.6% and 0.7%, respectively, thereby further verifying the capacity of these probiotics to suppress the colonization of conditional pathogens.

At the phylum level, the murine gut microbiota was predominantly composed of *Bacteroidetes* and *Firmicutes* (Figure 5D). The AD pathological state induced significant structural dysbiosis, characterized by a relative enrichment of Firmicutes and a depletion of *Bacteroidetes*. Quantitative analysis revealed that the *Bacteroidetes* to *Firmicutes* (B/F) ratio in the AD model group significantly decreased to 0.41 ± 0.11, which was markedly lower than that of the BC group (0.66 ± 0.14). Following intervention, microbial homeostasis was restored to varying degrees: the B/F ratio increased to 0.57 ± 0.13 in the LA4 group, 0.58 ± 0.13 in the F5 group, and 0.50 ± 0.12 in the PC group.

Linear discriminant analysis Effect Size (LEfSe) (LDA score > 3.8) was employed to further identify key differentially abundant taxa across groups (Figure 5F). The characteristic microbiota of the model group was primarily concentrated in *Clostridium*, *Lachnospiraceae*, *Campylobacter*, and *Helicobacter*, suggesting an enrichment of harmful bacterial populations. In contrast, *Dubosiella* and *Erysipelotrichaceae* were dominant in the *Lb. acidophilus* LA4 group, whereas *Lactobacilli* were the primary characteristic genus in the *Ls. paracasei* F5 group.

In conclusion, the structural improvements in the gut microecosystem induced by probiotic intervention, particularly the restoration of SCFAs producing bacteria and the suppression of pathogens, are highly consistent with the previously observed alleviation of neuroinflammation, reduction in Aβ deposition, and enhancement of cognitive function. These findings provide direct microbiological evidence that probiotics remodel bidirectional gut–brain communication via the “microbiota-immune-neuro” axis, thereby intervening in the pathological processes of the central nervous system.

### 3.6. Untargeted Metabolomics Analysis of Gut Contents

**Figure 6:**
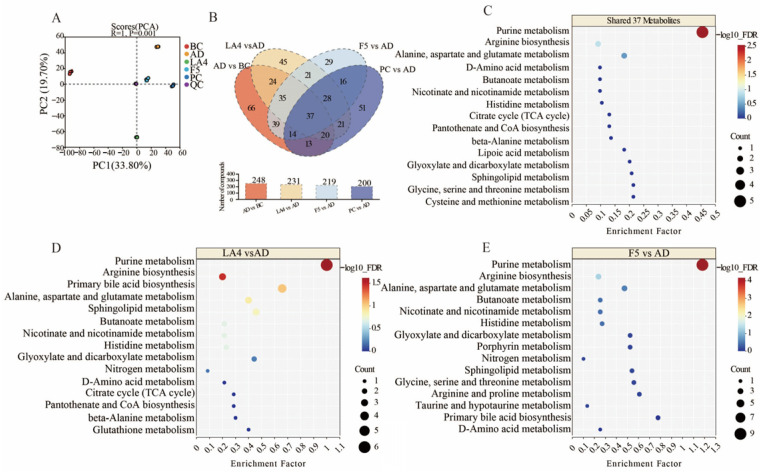
Untargeted metabolomic analysis of colonic contents in each group. (**A**) PCA score plot of metabolite distributions. (**B**) Venn diagram showing overlapping differential metabolites between LA4, F5, and PC groups relative to the AD group. (**C**–**E**) Bubble plots of KEGG pathway enrichment for (**C**) the 37 shared metabolites, (**D**) LA4-specific effects, and (**E**) F5-specific effects. The size of the bubbles represents the count of metabolites, and the color represents the −log_10_FDR.

Untargeted metabolomics was employed to evaluate the impact of probiotic intervention on the colonic metabolic profiles of AD mice. The Principal Component Analysis (PCA) score plot (Figure 6A) revealed clear clustering and distinct separation among the BC, AD, LA4, F5, and PC groups. The first and second principal components (PC1 and PC2) accounted for 33.80% and 19.70% of the variance, respectively. This distinct separation indicates that the colonic metabolic patterns in AD mice were altered and that probiotic intervention exerted a regulatory effect on these metabolic profiles. Differential metabolites were identified based on multidimensional statistical screening criteria, specifically requiring a variable importance in projection (VIP) score > 1 and *p* < 0.05 derived from the PLS-DA model. Results showed that 231, 219, and 200 differential metabolites were identified in the LA4, F5, and PC groups, respectively, compared to the AD group.

Venn diagram analysis (Figure 6B) revealed both commonalities and specificities in how different probiotic strains modulated the intestinal microenvironment of AD mice. A total of 37 key differential metabolites were shared across all intervention groups, suggesting that despite strain differences, there is a core synergistic action in remodeling the disordered intestinal metabolic network associated with AD. Pathway enrichment analysis of these 37 shared metabolites using MetaboAnalyst 5.0 [53] demonstrated significant co-regulation of purine metabolism, arginine biosynthesis, and alanine, aspartate, and glutamate metabolism (Figure 6C). The “−log_10_(FDR)” scale (FDR: False Discovery Rate, estimated using the Benjamini–Hochberg procedure [54]) indicates the statistical significance of pathway enrichment, with higher values denoting greater significance. Alterations in these core pathways may be closely related to energy metabolism disorders and neurotransmitter imbalances inherent to AD pathology. Existing literature indicates that disorders in purine metabolism not only affect cellular energy supply but are also tightly linked to AD-associated inflammatory responses; regulating purine metabolism and the gut microbiota-metabolite homeostasis has been reported to effectively improve learning and memory capabilities and alleviate brain pathology in AD model mice [55].

Further strain-specific analysis revealed unique strategies for metabolic reprogramming employed by LA4 and F5. In addition to regulating the aforementioned shared pathways, the LA4 group significantly influenced primary bile acid biosynthesis and sphingolipid metabolism (Figure 6D). Bile acids serve as critical signaling molecules communicating along the gut–brain axis, and alterations in their metabolic profiles are directly correlated with neuroinflammation levels in AD. Previous studies have noted that changes in gut microbiota composition can hinder the production of key metabolites, including secondary bile acids, thereby influencing behavioral deficits and neuroinflammatory cascades in mice [56,57]. Furthermore, the regulation of sphingolipid metabolism suggests that LA4 may exert neuroprotection via lipid signaling. Literature details how microbial metabolites, particularly sphingolipids and phospholipids, can act as core components of the gut–brain axis, modulating AD pathological mechanisms such as β-amyloid metabolism and Tau phosphorylation [19]. This suggests that LA4 may inhibit neuroinflammation by specifically repairing lipid metabolic networks and bile acid signaling.

In contrast, the F5 group exhibited significant regulatory effects on butanoate metabolism and nicotinate and nicotinamide metabolism (Figure 6E). Butyrate, a representative SCFAs, is a key maintainer of intestinal barrier integrity and a potent anti-inflammatory agent. Research has confirmed that regulating the microbiota and the metabolite butyrate can significantly attenuate microglia-mediated neuroinflammation, a finding highly consistent with the significant enrichment of the butyrate metabolism pathway observed in the F5 group [58]. Additionally, the regulation of nicotinate and nicotinamide metabolism is directly linked to NAD+ biosynthesis and mitochondrial function. The imbalance of NAD+/NADH metabolism plays a central role in AD oxidative stress and pathogenesis [59]. Therefore, F5 may exert its specific neuroprotective effects through a strategy of enhancing the anti-inflammatory action of SCFAs and improving mitochondrial energy metabolism.

In summary, while both LA4 and F5 ameliorated AD-associated metabolic dysbiosis, LA4 tended to maintain neuronal homeostasis by regulating lipid and bile acid signals, whereas F5 focused more on exerting effects through SCFAs and energy metabolism pathways. These strain-specific metabolic regulatory mechanisms provide an important theoretical basis for the development of precision probiotic therapies for AD.

## 4. Discussion

AD is a complex neurodegenerative disorder with pathogenic mechanisms involving multiple pathological cascades, including Aβ deposition, Tau protein hyperphosphorylation, cholinergic deficits, and neuroinflammation. Current pharmacological treatments primarily offer symptomatic relief without reversing the disease course. This study aims to screen probiotic strains with neuroprotective potential using multimodal in vivo experimental models and to elucidate their molecular mechanisms underlying the alleviation of AD pathological features, as well as their role in modulating the gut microbiota. Probiotic strains *Lb. acidophilus* LA4, *Ls. paracasei* F, and *Ls. rhamnosus* LR were selected for preliminary screening using a *Drosophila* melanogaster model to identify candidates capable of alleviating AD related pathology. Based on these findings, the selected probiotic strains were subsequently evaluated in D-galactose/AlCl_3_ induced-mouse models of AD. The results indicate that *Lb. acidophilus* LA4 and *Ls. paracasei* F5 significantly improved cognitive impairment and alleviated cerebral pathological damage.

The employment of a multi-staged screening system, transitioning from *Drosophila* model to the complex physiological environment of mice, represents a significant methodological strength of this study. While *Drosophila* allows for the rapid identification of neuroprotective candidates by observing motor coordination and Aβ_42_ accumulation [60], the murine model provides a necessary platform to evaluate sophisticated cognitive behaviors and systemic metabolic shifts. Our findings that LA4 and F5 consistently alleviated AD-like pathologies across both species underscore the robustness of these strains’ neuroprotective efficacy. This cross-species consistency is particularly noteworthy, as it suggests that the underlying mechanisms, likely involving fundamental pathways of the microbiota-gut–brain axis, are evolutionary conserved [61]. This approach not only enhances the reliability of the results but also demonstrates a high-efficiency paradigm for screening functional food ingredients for neurodegenerative diseases.

This study found that intervention with LA4 and F5 significantly reduced the content of Aβ_1–42_ and the phosphorylation levels of Tau protein in the hippocampal tissue of AD mice, while concurrently inhibiting excessive AChE activity. The Aβ cascade and Tau pathology are core factors driving synaptic dysfunction and neuronal death. Reducing Aβ oligomers and fibrillar deposition represents a key strategy for disease-modifying therapies [62]. Our results suggest that probiotics may indirectly promote Aβ clearance or reduce its generation by attenuating peripheral and central inflammatory signals. Furthermore, the reduction in AChE activity contributes to the restoration of acetylcholine levels and the improvement of cholinergic transmission. This mechanism parallels the action of the positive control drug, donepezil, and further verifies the argument by Moss et al. [63] regarding the critical role of cholinergic protection in cognitive improvement.

Neuroinflammation and oxidative stress exhibit a reciprocal causal relationship, jointly driving the pathological progression of AD. This study showed that pro-inflammatory cytokines (TNF-*α*, IL-6, IL-1β) were significantly elevated in the brains of AD model mice, whereas intervention with LA4 and F5 effectively reversed this trend. These results suggest that probiotic intervention may confer neuroprotective effects by attenuating the excessive activation of neural cells. As emphasized by He et al. [64],microglial dysfunction plays a central role in AD neuroinflammation, and probiotic metabolites (such as SCFAs) are known to penetrate the blood–brain barrier and modulate microglial phenotypes. Concurrently, we observed decreased MDA levels and increased SOD and GSH-Px activities in the probiotic-treated groups, suggesting an enhancement of the antioxidant defense system. These results support the perspective of Kurhaluk et al. [65] that modulating redox balance via the gut–brain axis is an effective pathway for the prevention and treatment of neurodegenerative diseases.

Gut microbiota dysbiosis is a significant environmental factor in the pathogenesis of AD. Research indicates that dysbiosis is thought to contribute to early AD pathology by promoting immune aging, cytokine imbalances, and neuroinflammation [66]. In this study, AD mice exhibited distinct gut dysbiosis (characterized by a reduction in beneficial bacteria such as *Muribaculaceae* and an increase in pathogenic bacteria such as *Helicobacter*), whereas intervention with LA4 and F5 significantly restored microbial diversity and the homeostasis of the *Firmicutes/Bacteroidetes* (F/B) ratio. The observed alterations in *Muribacoccus* abundance, coupled with reduced levels of inflammatory cytokines, further corroborate the universality of the “probiotics-short-chain fatty acids-anti-inflammation–neuroprotection” axis [14,67].Changes in the abundance of specific gut microbiota are closely associated with cognitive decline in AD [68].

A key highlight of this study is the identification of strain-specific metabolic signatures. Untargeted metabolomics analysis revealed significant regulation of purine metabolism, arginine biosynthesis, and bile acid metabolic pathways by these probiotics. For instance, the LA4 group significantly influenced the primary bile acid biosynthesis pathway. Zhang et al. [19] elaborated that bile acids, acting as signaling molecules, can modulate blood–brain barrier permeability and neuroinflammation via receptor-mediated mechanisms. Furthermore, bile acids have been shown to be associated with increased Aβ production in AD [67,69]. Additionally, the enrichment of butyrate metabolism in the F5 group aligns with the findings of Sun et al. [58], in which butyrate exerts neuroprotection by inhibiting histone deacetylases (HDACs) or activating anti-inflammatory pathways. This divergence suggests that while the “take-home message” is one of synergistic alleviation of AD, the “entry points” for these strains differ. LA4 appears more focused on maintaining neuronal homeostasis via lipid signaling, while F5 targets energy metabolism and butyrate-mediated anti-inflammation.

The evidence above emphasizes the pivotal role of the gut microbiota and its metabolites in modulating neuroinflammatory processes, thereby influencing the pathogenesis and progression of AD. Disruption of the gut–brain axis, particularly through increased intestinal and blood–brain barrier permeability, enables the translocation of harmful microbial metabolites into the central nervous system, where they significantly contribute to neuroinflammation. This chronic inflammatory state promotes the accumulation of amyloid-β plaques and hyperphosphorylated tau tangles, ultimately impairing neuronal function and accelerating AD progression.

Probiotic intervention has the potential to disrupt this vicious cycle. Colonizing probiotic strains restore gut microbial homeostasis, enhance the colonization of beneficial bacteria, and metabolize dietary fiber into SCFA-notably butyrate and propionate—while concurrently suppressing the production of harmful metabolites such as secondary bile acids. These actions collectively attenuate neuroinflammation, reduce Aβ deposition, and inhibit tau hyperphosphorylation, thereby presenting a safe, feasible, and sustainable strategy for early intervention in AD.

Moreover, although both *Lb. acidophilus* LA4 and *Ls. paracasei* F5 exhibited neuroprotective effects, with both demonstrating superior efficacy to *Ls. rhamnosus* LR, their specific therapeutic focuses differed slightly. LA4 was more prominent in alleviating anxiety-like behaviors and regulating sphingolipid and bile acid metabolism, which may be attributable to its specific genomic characteristics and metabolic products. Jouni et al. [70] have also reported strain-specific effects of different probiotics on the serum metabolic profiles of AD patients. This suggests that in future clinical applications, precise microecological interventions based on specific strains should be implemented according to the unique metabolic characteristics and pathological manifestations of patients.

It should be noted that while this study provides a robust functional assessment of LA4 and F5 across different species, the precise molecular cascading events remain to be fully elucidated. This work establishes a necessary foundation for future studies focusing on specific receptor-mediated signaling pathways in AD.

## 5. Conclusions

In summary, by establishing a dual-model system combining *Drosophila* melanogaster screening with mouse validation, we systematically evaluated the therapeutic effects of three lactic acid bacterial strains on AD-related pathological phenotypes. This integrated approach confirmed that *Lb. acidophilus* LA4 and *Ls. paracasei* F5 exhibit significant neuroprotective potential. These two probiotic strains attenuate AD progression through synergistic, multi-target mechanisms. At the behavioral level, they ameliorate cognitive deficits and anxiety-like behaviors in AD model animals. At the cerebral level, they improve cognitive function by inhibiting neuroinflammation, mitigating oxidative stress, and blocking Aβ and Tau pathologies. At the intestinal level, they improve internal environmental homeostasis by remodeling the microbiota structure and metabolic networks (e.g., bile acid and short-chain fatty acids pathways). This study provides a solid theoretical basis and data support for the use of these probiotics as an adjuvant therapeutic strategy for AD.

## 6. Patents

Liu, H.H.; Liu, J.; Liu, L.L.; Zhu, Q.M.; Sun, Z.O.; Zhao, Y.Q. *Lactobacillus acidophilus* LA4 and Its Applications. (In Chinese) Patent Application No. 202511246540.2.

## Data Availability

The original contributions presented in this study are included in the article/Supplementary Materials. Further inquiries can be directed to the corresponding authors.

## Supplementary Materials

The following supporting information can be downloaded at: https://www.mdpi.com/article/10.3390/foods15030429/s1, Figure S1. Phylogenetic analysis of 3 strains of probiotics based on 16S rDNA sequences. (A) *Lb. acidophilus* LA4, (B) *Ls. paracasei* F5, (C) *Ls. rhamnosus* LR.

## Abbreviations

The following abbreviations are used in this manuscript:

Aβ	Amyloid-beta
AChE	Acetylcholinesterase
AD	Alzheimer’s Disease
AlCl_3_	Aluminum Chloride
AMPK	AMP-activated protein kinase
Akt	Protein Kinase B
BC	Blank Control
CFU	Colony Forming Units
DAPI	4′,6-diamidino-2-phenylindole
D-gal	D-galactose
ELISA	Enzyme-Linked Immunosorbent Assay
FDR	False Discovery Rate
ESI	Electrospray Ionization
F5	*Lacticaseibacillus paracasei* F5
F/B	*Firmicutes* to *Bacteroidetes* ratio
GSH-Px	Glutathione Peroxidase
H&E	Hematoxylin and Eosin
HDAC	Histone Deacetylase
IL-1β	Interleukin-1 beta
IL-6	Interleukin-6
KEGG	Kyoto Encyclopedia of Genes and Genomes
LA4	*Lactobacillus acidophilus* LA4
LC-MS	Liquid Chromatography-Mass Spectrometry
LDA	Linear Discriminant Analysis
LEfSe	Linear Discriminant Analysis Effect Size
LR	*Lacticaseibacillus rhamnosus* LR
MDA	Malondialdehyde
MWM	Morris Water Maze
NFTs	Neurofibrillary Tangles
NMDA	N-methyl-D-aspartate
OFT	Open Field Test
OTU	Operational Taxonomic Unit
PC	Positive Control group
PCA	Principal Component Analysis
PCoA	Principal Coordinate Analysis
p-Tau	Phosphorylated Tau
SCFAs	Short-Chain Fatty Acids
SD	Standard Deviation
SOD	Superoxide Dismutase
TLR4	Toll-like Receptor 4
TNF-*α*	Tumor Necrosis Factor-alpha
UHPLC	Ultra-High Performance Liquid Chromatography
