# Supplementary material for: Probiotics Lactobacillus acidophilus LA4 and Lacticaseibacillus paracasei F5 Alleviate Cognitive Dysfunction in Alzheimer’s Disease Models: A Dual-Screening Study in Drosophila and Mice
Source: Foods. 2026 Jan 24;15(3):429. doi: 10.3390/foods15030429 (PMC12897270; doi:10.3390/foods15030429)
Supplement: Supplementary file 1 [file foods-15-00429-s001.zip › Figure S1.pdf]

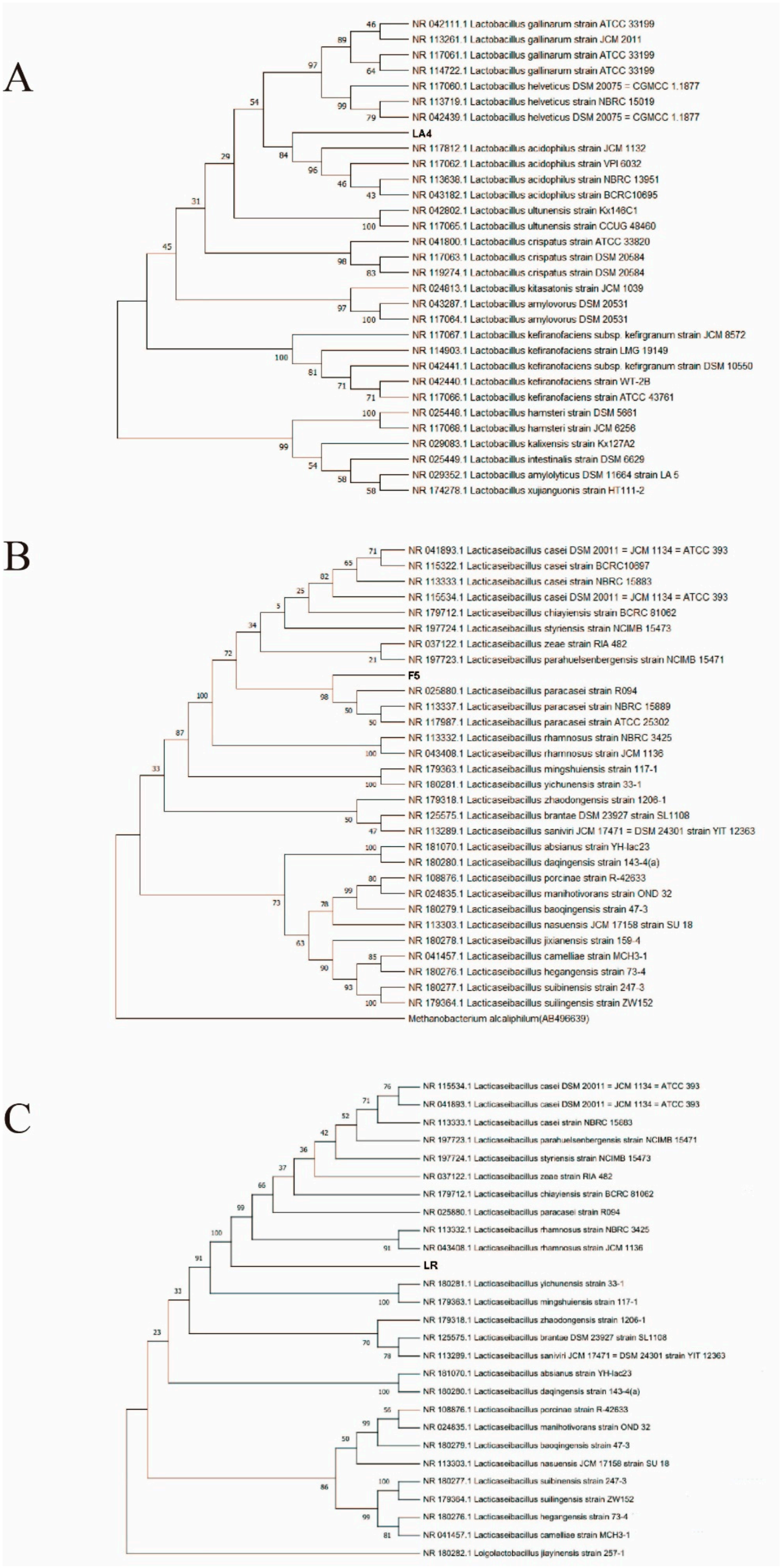

Figure S1. Phylogenetic analysis of 3 strains of probiotics based on 16S rDNA sequences.
(A) *Lb. acidophilus* LA4, (B) *Ls. paracasei* F5, (C) *Ls. rhamnosus* LR.
